# The Research Progress of PD-1/PD-L1 Inhibitors Enhancing Radiotherapy Efficacy

**DOI:** 10.3389/fonc.2021.799957

**Published:** 2021-12-09

**Authors:** Lu Wen, Fan Tong, Ruiguang Zhang, Lingjuan Chen, Yu Huang, Xiaorong Dong

**Affiliations:** Cancer Center, Union Hospital, Tongji Medical College, Huazhong University of Science and Technology, Wuhan, China

**Keywords:** radiotherapy, immunotherapy, PD-1/PD-L1, inhibitors, abscopal effect

## Abstract

Approximately 60%–70% of patients with malignant tumours require radiotherapy. The clinical application of immune checkpoint inhibitors (ICIs), such as anti-PD-1/PD-L1, has revolutionized cancer treatment and greatly improved the outcome of a variety of cancers by boosting host immunity.However, radiotherapy is a double-edged sword for PD-1/PD-L immunotherapy. Research on how to improve radiotherapy efficacy using PD-1/PD-L1 inhibitor is gaining momentum. Various studies have reported the survival benefits of the combined application of radiotherapy and PD-1/PD-L1 inhibitor. To fully exerts the immune activation effect of radiotherapy, while avoiding the immunosuppressive effect of radiotherapy as much as possible, the dose selection, segmentation mode, treatment timing and the number of treatment sites of radiotherapy play a role. Therefore, we aim to review the effect of radiotherapy combined with anti-PD-1/PD-L1 on the immune system and its optimization.

## Introduction

**Figure 1:**
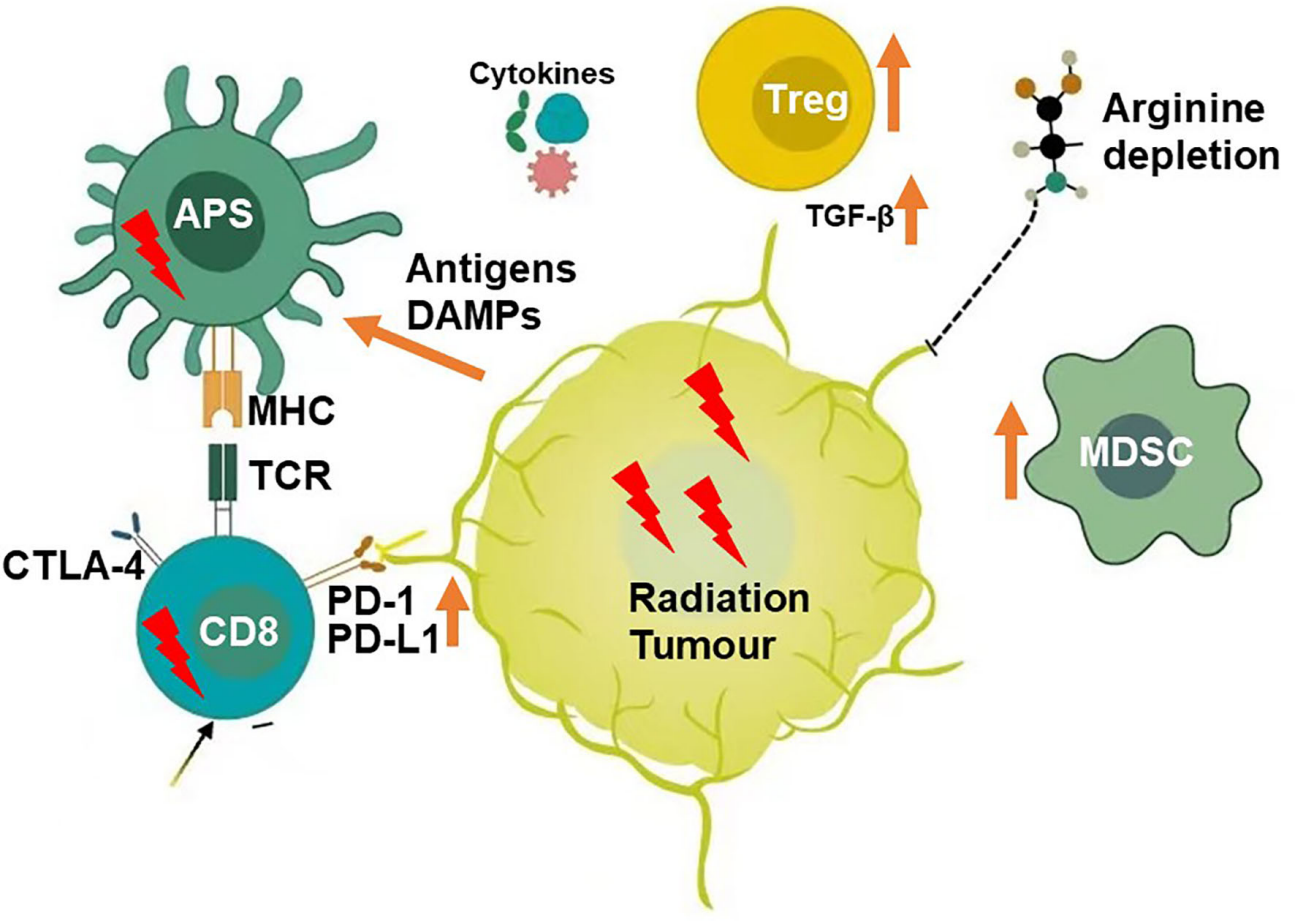
Microenvironment Modifiers(Radiation).

Radiotherapy is the main treatment option for tumours. Approximately 60%–70% of patients with malignant tumours require radiotherapy. The radiosensitivity of tumour cells is the key to its curative effect. Radiotherapy can directly act on tumour cell DNA, killing the cells, and it can also change the tumour microenvironment by producing *in situ* tumour vaccines that induce immune activation, triggering anti-tumour responses, and inducing the potential of tumour regression in non-irradiated areas, which is called abscopal effect (1–5). In some cases, the specific anti-tumour effect induced using radiotherapy is limited and radiotherapy efficacy is unsatisfactory. Additionally, while radiotherapy kills tumour cells, it can also damage the immune cells in the irradiated area. Therefore, radiotherapy is considered to be a double-edged sword. Radiotherapy can up-regulate PD-L1 expression and inhibit T cell activity (6). Radiotherapy can also activate the anti-tumour immune response (7–10). For example, radiotherapy can release a large number of tumour-related antigens by killing tumour cells, inducing an increase in tumour-infiltrating lymphocytes, and enhancing the anti-tumour immune response mediated by CD8+ T cells (9). Moreover, radiotherapy can promote the activation and maturation of dendritic cells. It also promotes antigen presentation by up-regulating MHC I expression tumour cell surface (7, 8). Therefore, it is feasible to combine radiotherapy and immunotherapy based on the immune-stimulating properties of radiotherapy.PD-1/PD-L1 inhibitor has been approved for the treatment of oesophageal, head and neck, melanoma, kidney, bladder, lung cancers and other tumours. Some tumours are treated with PD-1/PD-L1 inhibitor alone, which has good sensitivity and efficacy rate < 25% (11–33). Some tumours are almost ineffective, such as microsatellite stable colorectal cancer and EGFR (+) lung cancer (1, 34). The combined applications of PD-1/PD-L1 monoclonal antibody and radiotherapy have been reported to have good efficacy (35). Such combination therapies can enhance the body’s anti-tumour immune response and increase the abscopal effect on distant tumour inhibitions (36). However, when radiotherapy is combined with PD-1/PD-L1 treatment, it is necessary to consider the maximization of the immune activation effect of radiotherapy and avoidance of the immunosuppressive effect of radiotherapy. Therefore, the segmentation mode, dosage, combined action mechanism, and radiotherapy treatment part numbers need to be studied. For patients with multiple metastatic tumours, the practice of irradiating a single metastasis and expecting the abscopal effect should be abandoned. Instead, systemic therapy based on the PD-1/PD-L1 inhibitor and multi-site radiotherapy to enhance its efficacy should be considered (37). This article reviews the optimization of the combined PD-1/PD-L1 and radiotherapy treatment option (**Figure 1**).

## Mechanism of PD-1/PD-L1 Promoting Radiotherapy Efficacy

**Figure 2:**
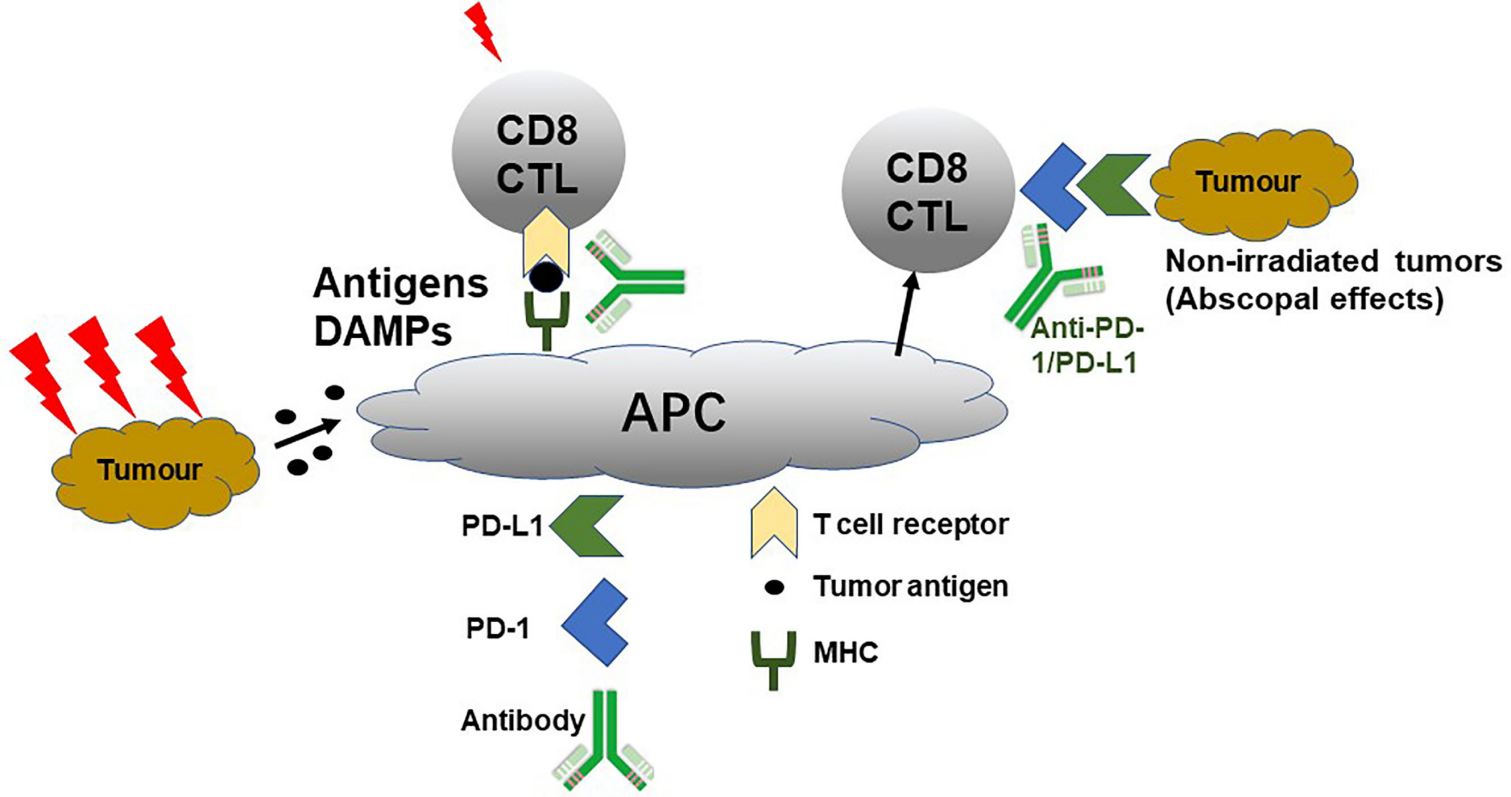
PD-1/PD-L1 Promoting Radiotherapy.

Clinically, immunotherapy can be combined with radiotherapy. Radiotherapy can induce tumour antigen release, enhance tumour cell immunogenicity, activate immune cells, secrete immune factors and promote tumour-related antigen presentation, and thereby effectively activating the anti-tumour immune response. Moreover, studies have shown that radiotherapy can up-regulate the expression of PD-1 on T cells and PD-L1 on tumour cells, leading to the inactivation and depletion of CD8+ T cells, suppression of immune responses and development of radiotherapy tolerance (38). If PD-1/PD-L1 monoclonal antibody is administered at the early stage of radiotherapy, it can restore T cell activity and enhance the anti-tumour immune response. Various studies have reported that TGF-β secretion increases after radiotherapy, leading to Treg infiltration and immune response suppression (39, 40). Radiotherapy combined with immunotherapy can reduce Treg numbers, increase CD8+T/Treg ratio and enhance tumour cell killings (41). Additionally, radiotherapy can promote HMGB1 release, stimulate calreticulin transportation to the cell surface and induce immunogenic cell death(ICD). Radiotherapy can increase protein breakdown, induce increased MHC I expression on the tumour cell surface and promote TAAs recognition by CTL cells. Radiotherapy causes tumour cell death, inducing the release of DAMPs, TAAs and inflammatory cytokines in cell debris and activating antigen-presenting cells, such as dendritic cells, to present TAAs to immune cells in the lymph nodes. Therefore, combined immunotherapy can enhance the radiotherapeutic immune induction and cooperates with radiotherapy to inhibit tumour growth, achieving an effect of 1 + 1 > 2 (42, 43). Additionally, radiotherapy also plays various roles in combination therapy for tumours of different stages and types. When the tumour burden is small and limited, radiotherapy can be used as a local radical treatment, aiming to cooperate with a systemic PD-1 inhibitor for curing cancer. In other cases, such as massive metastases or multiple metastatic tumours, radiotherapy can be used as an adjuvant for PD-1/PD-L1 inhibitors. Therefore, the combination of radiotherapy and PD-1/PD-L1inhibitor is diversified (**Figure 2**).

## Efficacy of Different Combinations of Radiotherapy and PD-1/PD-L1

The dose and division mode of radiotherapy combined with PD-1/PD-L1 have attracted widespread attention. Precise irradiation using hypofractionated radiotherapy (HFRT) can minimize the damage to the surrounding normal tissues. HFRT can induce a stronger immune response and abscopal effect than conventional radiotherapy. It is more suitable for combining with immunotherapy, and this theory has been confirmed by various studies. In 2020, Professor Lu You reported that HFRT can induce stronger local and systemic anti-tumour immune effects than conventional fractionated radiation therapy (CFRT) by inhibiting the VEGF/VEGFR signalling pathway, reducing MDSCs recruitment to the tumour microenvironment, mediating lower PD-L1 expression, decreasing tumour cells for immune escape through the PD-L1/PD-1 axis, increasing CD8+ T cell levels around tumour tissues and in peripheral blood and maintaining their tumour cell killing activity (44). Studies have further reported that HFRT combined with anti-PD-L1 antibody therapy can significantly improve the tumour (local and radiotherapy field lesions) control rate and survival rate in tumour-bearing mice (44). Clinically, the increase in Tregs affects the local control at 15 Gy/F and 1 F. The 7.5~10.0 Gy/F, 2~3 F regimen can maintain a low level of Treg, and it can better stimulate the body’s immune response safely (45, 46). The PEMBRO-RT study used 8 Gy/F, 3 F mode combined with PD-1 inhibitor to treat advanced non-small cell lung cancer (NSCLC). The ORR rate at 12-weeks was 36%, which increased more than once, compared with the control group’s progression-free survival (PFS) and overall survival (OS) rates (47). But, McBride et al. shown no improvement in response and no evidence of an abscopal effect with the addition of SBRT to nivolumab in unselected patients with metastatic HNSCC. Although the efficiency of immunotherapy may be improved by combined with radiotherapy, but the ORR of PD-1 antibody monotherapy for HNSCC is high, so it is necessary to further expand the sample size to reflect the difference between the experimental and control groups.

A consensus is yet to be reached over the optimal timing for the use of the combination therapy of radiotherapy and PD-1/PD-L1 inhibitor. Dovedi et al. found that simultaneous administration of anti-PD-1/PD-L1 antibody with conventional split RT has a higher survival rate than that of sequential administration (38). A phase I clinical study for advanced metastatic urothelial carcinoma reported that the effect of the simultaneous treatment group using PD-1 inhibitor receiving 8 Gy/F, 3F stereotactic body radiation therapy (SBRT) before the third cycle was significantly better than that of the sequential treatment group (48). The COSINR Phase I trial evaluates the simultaneous or sequential application of CTLA-4 inhibitor, PD-1 inhibitor and SBRT in patients with stage IV NSCLC. The trial’s latest data showed that the median PFS period of the sequential and contemporaneous groups was 5.9 and6.2 months, respectively (49). However, a study by Herter-Sprie et al. reported that the OS was similar to that of sequential administration (PD-1 antibody administration on the 7^th^ day after radiotherapy) regardless of the simultaneous administration from either the 1^st^ or 5^th^ day of radiotherapy (50). Some preclinical studies report contradictory results on the simultaneous or sequential use of radiotherapy combined with PD-1/PD-L1 inhibitor; however, an incline towards simultaneous use is observed. The subgroup analysis of the PACIFIC study showed that the PFS benefit trend of receiving PD-L1 monoclonal antibody within 14 days after concurrent radiotherapy and chemotherapy for stage III unresectable NSCLC was more significant than that of receiving PD-L1 antibody treatment for14 days (51–53). Radiotherapy combined with immune checkpoint inhibitors (ICIs) in patients with melanoma brain metastases showed that the combined ICI therapy within 4 weeks after treatment with stereotactic radiosurgery (SRS) had significantly better results than those in patients with SRS > 4 weeks (54). Evidence indicates that sequential treatment and PD-1/PD-L1 inhibitor administration after radiotherapy can increase the clinical benefit.

The timing of radiotherapy combined with anti-PD-1/PD-L1 therapy is affected by adverse effects along with therapeutic effectiveness. In the MDACC study, two patients with simultaneous HFRT or SBRT combined with PD-1 inhibitor had Grade 4 adverse effects, which may be attributed to the simultaneous medication. A study by ESMO 2020 showed that the administration of anti-PD-1 drugs before or during radiotherapy for thoracic tumours increased the incidence of radiation pneumonitis (60% 28%, P = 0.01) compared with the administration of anti-PD-1 drugs after radiotherapy (55, 56). However, the occurrence of adverse effects is closely related to factors such as radiotherapy dose, volume and location. Therefore, whether synchronization will increase adverse reactions than sequential treatment needs to be further confirmed by clinical studies.

For multiple metastatic tumours, there have been no large randomized controlled data on the number of lesions irradiated for the generation of the greatest monosensitization effect. Current methods include the partial irradiation of large tumours (46, 57), SBRT combined with low-dose irradiation (58, 59) and multiple periodic irradiations of different metastatic lesions. You et al. proposed for the first time a combination group of primary tumours receiving HFRT and secondary tumours receiving low-dose radiation therapy (LDRT), combined with ICIs. Compared with HFRT alone, secondary tumour growth in mice receiving LDRT combined treatment showed a significant decline in growth. LDRT strongly promotes the local infiltration of T cells into tumours and induces the lower recruitment of MDSCs; however, LDRT also promotes the up-regulation of immune activation related gene expression (antigen presentation related genes and T cell activation related genes) and T cell-related chemokine expression. It has also been confirmed in mouse CT26 and MC38 colon cancer models that the triple treatment group achieved the best secondary tumour growth control. Therefore, it has been proposed that the secondary tumour receiving LDRT can promote the migration of effector T cells into the tumour, reshape the local tumour microenvironment, amplify the abscopal effect of HFRT and increase the efficacy of combined immunotherapy.

## Radiotherapy Combined With Immunotherapy Increase Abscopal Effect

The abscopal effect was proposed by Mole in 1953. The abscopal effect is achieved through the activation of the immune system, which may be closely related to the increase in T cell activating factors, increase in existing tumour-specific antibodies and formation of new anti-tumour antibodies (60) The production of the abscopal effect by radiotherapy alone is rare in clinical practice and has been reported only in a few individual cases. Recently, radiotherapy combined with immunotherapy has caused a significant increase in the abscopal effect, but the mechanism of action remains unclear. Studies have found that PD-1/PD-L1 monoclonal antibody combined with radiotherapy can inhibit distant tumours through the abscopal effect. A study of melanoma (B16-OVA) mice reported that PD-1 monoclonal antibody combined with Stereotactic ablative brachytherapy (SABT) can decrease the primary lesion close to CR, and the tumour volume at the non-irradiated site is also reduced by 66% (61–63). SBRT can easily induce the abscopal effect compared to the conventional segmentation mode and is more suited for combining with immunotherapy (64). Attesting to this, Deng et al. reported that breast cancer mice receiving radiotherapy combined with PD-L1 monoclonal antibody treatment showed a reduction in the volume of distant tumours outside the radiotherapy site, and thereby producing a lasting immune memory (65). You et al. reported, for the first time, that HFRT induces primary tumour cell apoptosis, produces an “*in situ* vaccination” effect and sensitizes tumour-specific T cells. Moreover, LDRT promotes the migration of tumour-specific T cells into the secondary tumour. The combination of these two therapies (HFRT and LDRT) produces CD8+T cell-dependent immune effects. Meanwhile, PD-1 inhibitor restores the tumour-killing activity of T cells by releasing the “inhibitory brake” on the surface of T cells, further enhancing the systemic anti-tumour immune effect (58). Additionally, the results from the 2015 “Lancet” clinical trial on local radiotherapy combined with immunotherapy confirmed the production of the abscopal effect in approximately a quarter of patients with advanced tumours (including NSCLC, breast cancer and thymic cancer). Patients producing the abscopal effect had more obvious survival rates (60, 66).

## Alternate Methods to Enhance Radiotherapy Efficacy

Enhancement of the radiotherapy-related anti-tumour immune response can be performed *via* various methods as elucidated by the reports on the treatment mode of PD-L1 and CTLA-4 monoclonal antibodies combined with radiotherapy (67). In melanoma mice receiving radiotherapy combined with CTLA-4 monoclonal antibody, the tumour cell PD-L1 expression was significantly up-regulated. Therefore, the combination of PD-L1 monoclonal antibodies can restore the function of T cells, increase the ratio of CD8+T/Treg and increase the CR rate of mice to 80%. This study aims to reveal that triple therapy synergistically enhances the anti-tumour effects. Additionally, IL-2, granulocyte-macrophage colony-stimulating factor (GM-CSF), interferon-α and tumour necrosis factor-α (TNF-α) participate at different steps in the synergistic effect of radiotherapy and immunotherapy on tumour cells. IL-2 can promote the proliferation and activation of T cells and also activate NK cells. Radiotherapy can up-regulate the expression of MHC-I molecules and promote the formation of memory T cells. In the models of mouse melanoma, colon cancer and breast cancer, HFRT combined with IL-2 complex can produce a significant synergistic effect, enhancing the anti-tumour effects of CD8+ T and NK cells (68). Phase I clinical studies have reported that combining SBRT with IL-2 for the treatment of metastatic renal cell carcinoma and melanoma has a remission rate of 66. 6%. The response rate of melanoma was 71.4% (69, 70). Moreover, the combination of IL-2 and radiotherapy can synergistically control the combined treatment of local and distant lesions (69–73). GM-CSF promotes monocytes/M1 macrophage and DC differentiation, enhances antigen presentation and amplifies the body’s immune effect (74). A clinical trial has reported that local radiotherapy combined with GM-CSF subcutaneous injection induced the abscopal effect at a rate of 22.2% in NSCLC and OS showed significant prolongation (60). A clinical study of patients with advanced cholangiocarcinoma who received PD-1 inhibitor combined with GM-CSF showed that the 6-month PFS rate reached 35% (75). A prospective clinical study of single-arm HFRT combined with PD-1 inhibitor and GM-CSF in the treatment of advanced multiple metastatic solid tumours is being carried out. The median PFS stage is 4.0 months. The current study is still in progress (ChiCTR1900026175) (76).

TNF-α is produced by activated macrophages and can induce immune cell activation. A phase I clinical study found that the combined treatment of TNF-α and radiotherapy improved OS and PFS in oesophageal cancer, head and neck cancer and other solid tumours (77).

## Conclusion

Immunotherapy has a dramatic impact on the field of oncology. Many pre-clinical data show that radiotherapy combined with immunotherapy enhances tumour killing through the vaccine effect, attraction effect and fragility effect. The synergistic effects of PD-1 and PD-L1 monoclonal antibodies combined with radiotherapy have been confirmed in various preclinical trials. Such combination therapies can enhance the body’s anti-tumour immune response and increase the abscopal effect on distant tumour inhibitions. However, this treatment model is still in its infancy, it is necessary to consider the maximization of the immune activation effect of radiotherapy and avoidance of the immunosuppressive effect of radiotherapy (78). Radiotherapy dose, segmentation method, irradiation site, radiotherapy volume, intervention point of immunotherapy, selection of immunodrugs and disease/patient all need to be demonstrated by more sufficient clinical trial data. There are many clinical studies at home and abroad that are actively trying to add radiotherapy to various immunotherapy strategies to determine the best therapy combination (**Table 1**). Further studies on answering the current problems in combination therapy and making radiotherapy combined with immunotherapy clinically effective are required.

**Table 1:** Immunotherapy agents under clinical investigation in combination with radiation.

Category	Reagent	Diseases	No. of current studies
Checkpoint inhibitors	Anti-CTLA-4	Cervix, melanoma, head and neck, NSCLC, pancreas, liver, lung, Breast, colon	100
	PD-1/PD-L-1	Esophageal, NSCLC, Malignant glioma, melanoma (brain metastases), invasive bladder, oligometastatic breast, head and neck, pancreas, gastric, colorectal, follicular lymphoma, Extensive Stage Small Cell Lung, Prostate, urothelial, Gastroensophageal, HCC, Pancreatic, renal, colon, glioblastoma	
Vaccines/oncolytic Viruses	AdV-tk, Sipluleucel-T, G207, ADV/HSV-tk, Oncolytic Adenovirus Ad5-yCD/mutTKSR39rep-hIL12, and Ad5-yCD/MutTKSR39rep-AD	Prostate, pancreas, malignant supratentorial neoplasms, NSCLC, triple negative breast, prostate, glioma, ovarian, sarcoma, glioblastoma, oesophageal	28
Cytokines	IL-2, IFN, GM-CSF, and TGF-beta blockade	Metastatic breast, NSCLC, glioblastoma, follicular lymphoma, and pancreas, renal, advanced hepacellular carcinoma, oesophageal, colorectal, melanoma, glioma	155
Other targeted immune Rx	OX40 antibody, CDX-301, GITR, and TLR-4,7,9 agonists	Melanoma, renal cell carcinoma, NSCLC, breast, sarcoma, cutaneous T-cell and recurrent lymphoma, breast, colorectal, fibrosarcoma, fibrosarcoma, lung, melanoma, osteosarcoma, renal, B-cell lymphomas	27

## Author Contributions

LW is the main writer of this review. FT and RZ participate in the analysis and sorting of literature data. LC and YH complete the collection and analysis of relevant literature data. XD conceptulize this review. All authors contributed to the article and approved the submitted version.

## Funding

This research was supported by the grants from the National Key R&D Program of China (Grant No 2019YFC1316205).

## Conflict of Interest

The authors declare that the research was conducted in the absence of any commercial or financial relationships that could be construed as a potential conflict of interest.

## Publisher’s Note

All claims expressed in this article are solely those of the authors and do not necessarily represent those of their affiliated organizations, or those of the publisher, the editors and the reviewers. Any product that may be evaluated in this article, or claim that may be made by its manufacturer, is not guaranteed or endorsed by the publisher.

## References

[1] Bernstein MB Krishnan S Hodge JW Chang JY. Immunotherapy and Stereotactic Ablative Radiotherapy (ISABR): A Curative Approach? Nat Rev Clin Oncol (2016) 13(8):516–24. doi: 10.1038/nrclinonc.2016.30 PMC6053911 26951040

[2] Benson KRK Sandhu N Zhang C Ko R Toesca DAS Lee PE. Local Recurrence Outcomes of Colorectal Cancer Oligometastases Treated With Stereotactic Ablative Radiotherapy. Am J Clin Oncol (2021) 44:559–64. doi: 10.1097/COC.0000000000000864 34534143

[3] Chang JY Mehran RJ Feng L Verma V Liao Z Welsh JW. Stereotactic Ablative Radiotherapy for Operable Stage I Non-Small-Cell Lung Cancer (Revised STARS): Long-Term Results of a Single-Arm, Prospective Trial With Prespecified Comparison to Surgery. Lancet Oncol (2021)22(10):1448–57. doi: 10.1016/S1470-2045(21)00401-0 PMC8521627 34529930

[4] Chen X Chen H Poon I Erler D Badellino S Biswas T. Late Metastatic Presentation Is Associated With Improved Survival and Delayed Wide-Spread Progression After Ablative Stereotactic Body Radiotherapy for Oligometastasis. Cancer Med (2021) 10(18):6189–98. doi: 10.1002/cam4.4133 PMC8446561 34432390

[5] Chen Y Dai J Jiang Y Ji Z Jiang P Sun H. Long-Term Outcomes of Personalized Stereotactic Ablative Brachytherapy for Recurrent Head and Neck Adenoid Cystic Carcinoma After Surgery or External Beam Radiotherapy: A 9-Year Study. J Pers Med (2021) 11(9):839. doi: 10.3390/jpm11090839 34575616 PMC8467951

[6] Sato H Niimi A Yasuhara T Permata TBM Hagiwara Y Isono M. DNA Double-Strand Break Repair Pathway Regulates PD-L1 Expression in Cancer Cells. Nat Commun (2017) 8(1):1751. doi: 10.1038/s41467-017-01883-9 29170499 PMC5701012

[7] Reits EA Hodge JW Herberts CA Groothuis TA Chakraborty M Wansley EK. Radiation Modulates the Peptide Repertoire, Enhances MHC Class I Expression, and Induces Successful Antitumor Immunotherapy. J Exp Med (2006) 203(5):1259–71. doi: 10.1084/jem.20052494 PMC3212727 16636135

[8] Gupta A Probst HC Vuong V Landshammer A Muth S Yagita H. Radiotherapy Promotes Tumor-Specific Effector CD8+ T Cells via Dendritic Cell Activation. J Immunol (2012) 189(2):558–66. doi: 10.4049/jimmunol.1200563 22685313

[9] Lee Y Auh SL Wang Y Burnette B Wang Y Meng Y. Therapeutic Effects of Ablative Radiation on Local Tumor Require CD8+ T Cells: Changing Strategies for Cancer Treatment. Blood (2009) 114(3):589–95. doi: 10.1182/blood-2009-02-206870 PMC2713472 19349616

[10] Chakraborty M Abrams SI Camphausen K Liu K Scott T Coleman CN. Irradiation of Tumor Cells Up-Regulates Fas and Enhances CTL Lytic Activity and CTL Adoptive Immunotherapy. J Immunol (2003) 170(12):6338–47. doi: 10.4049/jimmunol.170.12.6338 12794167

[11] Guo W Zhang F Shao F Wang P Li Z Yang X. PD-L1 Expression on Tumor Cells Associated With Favorable Prognosis in Surgically Resected Esophageal Squamous Cell Carcinoma. Hum Pathol (2019) 84:291–8. doi: 10.1016/j.humpath.2018.09.014 30296523

[12] Jiang C Zhu Y Tang S Zhang G Lin Q Xu Y. High PD-L1 Expression Is Associated With a Favorable Prognosis in Patients With Esophageal Squamous Cell Carcinoma Undergoing Postoperative Adjuvant Radiotherapy. Oncol Lett (2019) 17(2):1626–34. doi: 10.3892/ol.2018.9747 PMC6341902 30675222

[13] Zayac A Almhanna K. Esophageal, Gastric Cancer and Immunotherapy: Small Steps in the Right Direction? Transl Gastroenterol Hepatol (2020) 5:9. doi: 10.21037/tgh.2019.09.05 32190777 PMC7061183

[14] Bu LL Yu GT Wu L Mao L Deng WW Liu JF. STAT3 Induces Immunosuppression by Upregulating PD-1/PD-L1 in HNSCC. J Dent Res (2017) 96(9):1027–34. doi: 10.1177/0022034517712435 PMC6728673 28605599

[15] Mann JE Hoesli R Michmerhuizen NL Devenport SN Ludwig ML Vandenberg TR. Surveilling the Potential for Precision Medicine-Driven PD-1/PD-L1-Targeted Therapy in HNSCC. J Cancer (2017) 8(3):332–44. doi: 10.7150/jca.17547 PMC5332883 28261333

[16] Gide TN Pires da Silva I Quek C Ferguson PM Batten M Shang P. Clinical and Molecular Heterogeneity in Patients With Innate Resistance to Anti-PD-1 +/- Anti-CTLA-4 Immunotherapy in Metastatic Melanoma Reveals Distinct Therapeutic Targets. Cancers (Basel) (2021) 13(13):3186. doi: 10.3390/cancers13133186 34202352 PMC8267740

[17] Liu Y Zhang X Wang G Cui X. Triple Combination Therapy With PD-1/PD-L1, BRAF, and MEK Inhibitor for Stage III-IV Melanoma: A Systematic Review and Meta-Analysis. Front Oncol (2021) 11:693655. doi: 10.3389/fonc.2021.693655 34195094 PMC8236832

[18] Barata P Hatton W Desai A Koshkin V Jaeger E Manogue C. Outcomes With First-Line PD-1/PD-L1 Inhibitor Monotherapy for Metastatic Renal Cell Carcinoma (mRCC): A Multi-Institutional Cohort. Front Oncol (2020) 10:581189. doi: 10.3389/fonc.2020.581189 33194712 PMC7642690

[19] Carril-Ajuria L Lora D Carretero-Gonzalez A Martin-Soberon M Rioja-Viera P Castellano D. Systemic Analysis and Review of Nivolumab-Ipilimumab Combination as a Rescue Strategy for Renal Cell Carcinoma After Treatment With Anti-PD-1/PD-L1 Therapy. Clin Genitourin Cancer (2021) 19(2):95–102. doi: 10.1016/j.clgc.2020.10.004 33189597

[20] Curran CS Kopp JB. PD-1 Immunobiology in Glomerulonephritis and Renal Cell Carcinoma. BMC Nephrol (2021) 22(1):80. doi: 10.1186/s12882-021-02257-6 33676416 PMC7936245

[21] Ding L Dong HY Zhou TR Wang YH Yan T Li JC. PD-1/PD-L1 Inhibitors-Based Treatment for Advanced Renal Cell Carcinoma: Mechanisms Affecting Efficacy and Combination Therapies. Cancer Med (2021) 10(18):6384–401. doi: 10.1002/cam4.4190 PMC8446416 34382349

[22] Huang J Wang Y Zhang H Hu X Wang P Cai W. Clinical Outcomes of Second-Line Treatment Following First-Line VEGFR-TKI Failure in Patients With Metastatic Renal Cell Carcinoma: A Comparison of Axitinib Alone and Axitinib Plus Anti-PD-1 Antibody. Cancer Commun (Lond) (2021) 41(10):1071–4. doi: 10.1002/cac2.12206 PMC8504141 34363742

[23] Hwang I Park I Yoon SK Lee JL. Clinical Course of Patients With Renal Cell Carcinoma or Urothelial Carcinoma Who had Stable Disease as an Initial Response to a PD-1 or PD-L1 Inhibitor. Asia Pac J Clin Oncol (2021). doi: 10.1111/ajco.13601 34219393

[24] Shiuan E Reddy A Dudzinski SO Lim AR Sugiura A Hongo R. Clinical Features and Multiplatform Molecular Analysis Assist in Understanding Patient Response to Anti-PD-1/PD-L1 in Renal Cell Carcinoma. Cancers (Basel) (2021) 13(6):1475. doi: 10.3390/cancers13061475 33806963 PMC8004696

[25] Hou W Xue M Shi J Yang M Zhong W Fan X. PD-1 Topographically Defines Distinct T Cell Subpopulations in Urothelial Cell Carcinoma of the Bladder and Predicts Patient Survival. Urol Oncol (2020) 38(8):685.e1–10. doi: 10.1016/j.urolonc.2020.04.009 32409198

[26] Wang J Li Q Lv H Nie C Chen B Xu W. A PD-1 Inhibitor Induces Complete Response of Advanced Bladder Urothelial Carcinoma: A Case Report. Front Oncol (2021) 11:671416. doi: 10.3389/fonc.2021.671416 34221988 PMC8249845

[27] Chen Q Li Y Zhang W Yang S Wang C Guo Q. Clinical Analysis of Docetaxel Combined With PD-1/PD-L1 Inhibitor in Second-Line Treatment of Advanced Non-Small Cell Lung Cancer. Zhongguo Fei Ai Za Zhi (2021) 24(9):605–12. doi: 10.3779/j.issn.1009-3419.2021.102.26 PMC8503983 34455735

[28] Geiger-Gritsch S Olschewski H Kocher F Wurm R Absenger G Flicker M. Real-World Experience With Anti-PD-1/PD-L1 Monotherapy in Patients With Non-Small Cell Lung Cancer: A Retrospective Austrian Multicenter Study. Wien Klin Wochenschr (2021) 133(21–22):1122–30. doi: 10.1007/s00508-021-01940-w 34528126

[29] Hamada K Yoshimura K Hirasawa Y Hosonuma M Murayama M Narikawa Y. Antibiotic Usage Reduced Overall Survival by Over 70% in Non-Small Cell Lung Cancer Patients on Anti-PD-1 Immunotherapy. Anticancer Res (2021) 41(10):4985–93. doi: 10.21873/anticanres.15312 34593446

[30] Leighl NB Redman MW Rizvi N Hirsch FR Mack PC Schwartz LH. Phase II Study of Durvalumab Plus Tremelimumab as Therapy for Patients With Previously Treated Anti-PD-1/PD-L1 Resistant Stage IV Squamous Cell Lung Cancer (Lung-MAP Substudy S1400F, Nct03373760). J Immunother Cancer (2021) 9(8):e002973. doi: 10.1136/jitc-2021-002973 34429332 PMC8386207

[31] Ni J Zhou Y Wang S Guo T Hu J Chu Q. A Brief Report on Incidence, Radiographic Feature and Prognostic Significance of Brain MRI Changes After Anti-PD-1/PD-L1 Therapy in Advanced Non-Small Cell Lung Cancer. Cancer Immunol Immunother (2021). doi: 10.1007/s00262-021-03070-8 PMC10991942 34613418

[32] Saar M Narits J Magi L Aaspollu H Vapper A Kase M. Expression of Immune Checkpoint PD-1 in Non-Small Cell Lung Cancer Is Associated With Tumor Cell DNA-Dependent Protein Kinase. Mol Clin Oncol (2021) 15(4):211. doi: 10.3892/mco.2021.2369 34462666 PMC8375025

[33] Sheng J Li H Yu X Yu S Chen K Pan G. Efficacy of PD-1/PD-L1 Inhibitors in Patients With Non-Small Cell Lung Cancer and Brain Metastases: A Real-World Retrospective Study in China. Thorac Cancer (2021) 12(22):3019–31. doi: 10.1111/1759-7714.14171 PMC8590903 34596346

[34] Yamamoto K Venida A Yano J Biancur DE Kakiuchi M Gupta S. Autophagy Promotes Immune Evasion of Pancreatic Cancer by Degrading MHC-I. Nature (2020) 581(7806):100–5. doi: 10.1038/s41586-020-2229-5 PMC7296553 32376951

[35] Teng F Kong L Meng X Yang J Yu J. Radiotherapy Combined With Immune Checkpoint Blockade Immunotherapy: Achievements and Challenges. Cancer Lett (2015) 365(1):23–9. doi: 10.1016/j.canlet.2015.05.012 25980820

[36] Park SS Dong H Liu X Harrington SM Krco CJ Grams MP. PD-1 Restrains Radiotherapy-Induced Abscopal Effect. Cancer Immunol Res (2015) 3(6):610–9. doi: 10.1158/2326-6066.CIR-14-0138 PMC4827718 25701325

[37] Brooks ED Chang JY. Time to Abandon Single-Site Irradiation for Inducing Abscopal Effects. Nat Rev Clin Oncol (2019) 16(2):123–35. doi: 10.1038/s41571-018-0119-7 30401936

[38] Dovedi SJ Adlard AL Lipowska-Bhalla G McKenna C Jones S Cheadle EJ. Acquired Resistance to Fractionated Radiotherapy Can Be Overcome by Concurrent PD-L1 Blockade. Cancer Res (2014) 74(19):5458–68. doi: 10.1158/0008-5472.CAN-14-1258 25274032

[39] Kachikwu EL Iwamoto KS Liao YP DeMarco JJ Agazaryan N Economou JS. Radiation Enhances Regulatory T Cell Representation. Int J Radiat Oncol Biol Phys (2011) 81(4):1128–35. doi: 10.1016/j.ijrobp.2010.09.034 PMC3117954 21093169

[40] Jonson CO Pihl M Nyholm C Cilio CM Ludvigsson J Faresjo M. Regulatory T Cell-Associated Activity in Photopheresis-Induced Immune Tolerance in Recent Onset Type 1 Diabetes Children. Clin Exp Immunol (2008) 153(2):174–81. doi: 10.1111/j.1365-2249.2008.03625.x PMC2492891 18549445

[41] Sharabi AB Nirschl CJ Kochel CM Nirschl TR Francica BJ Velarde E. Stereotactic Radiation Therapy Augments Antigen-Specific PD-1-Mediated Antitumor Immune Responses via Cross-Presentation of Tumor Antigen. Cancer Immunol Res (2015) 3(4):345–55. doi: 10.1158/2326-6066.CIR-14-0196 PMC4390444 25527358

[42] Perez-Gracia JL Labiano S Rodriguez-Ruiz ME Sanmamed MF Melero I. Orchestrating Immune Check-Point Blockade for Cancer Immunotherapy in Combinations. Curr Opin Immunol (2014) 27:89–97. doi: 10.1016/j.coi.2014.01.002 24485523

[43] Deng L Liang H Burnette B Weicheslbaum RR Fu YX. Radiation and Anti-PD-L1 Antibody Combinatorial Therapy Induces T Cell-Mediated Depletion of Myeloid-Derived Suppressor Cells and Tumor Regression. Oncoimmunology (2014) 3:e28499. doi: 10.4161/onci.28499 25050217 PMC4063144

[44] Lan J Li R Yin LM Deng L Gui J Chen BQ. Targeting Myeloid-Derived Suppressor Cells and Programmed Death Ligand 1 Confers Therapeutic Advantage of Ablative Hypofractionated Radiation Therapy Compared With Conventional Fractionated Radiation Therapy. Int J Radiat Oncol Biol Phys (2018) 101(1):74–87. doi: 10.1016/j.ijrobp.2018.01.071 29619980

[45] Schaue D Ratikan JA Iwamoto KS McBride WH. Maximizing Tumor Immunity With Fractionated Radiation. Int J Radiat Oncol Biol Phys (2012) 83(4):1306–10. doi: 10.1016/j.ijrobp.2011.09.049 PMC3337972 22208977

[46] Luke JJ Lemons JM Karrison TG Pitroda SP Melotek JM Zha Y. Safety and Clinical Activity of Pembrolizumab and Multisite Stereotactic Body Radiotherapy in Patients With Advanced Solid Tumors. J Clin Oncol (2018) 36(16):1611–8. doi: 10.1200/JCO.2017.76.2229 PMC5978468 29437535

[47] Theelen W Peulen HMU Lalezari F van der Noort V de Vries JF Aerts J. Effect of Pembrolizumab After Stereotactic Body Radiotherapy vs Pembrolizumab Alone on Tumor Response in Patients With Advanced Non-Small Cell Lung Cancer: Results of the PEMBRO-RT Phase 2 Randomized Clinical Trial. JAMA Oncol (2019) 5(9):1276–82. doi: 10.1001/jamaoncol.2019.1478 PMC6624814 31294749

[48] Sundahl N Vandekerkhove G Decaestecker K Meireson A De Visschere P Fonteyne V. Randomized Phase 1 Trial of Pembrolizumab With Sequential Versus Concomitant Stereotactic Body Radiotherapy in Metastatic Urothelial Carcinoma. Eur Urol (2019) 75(5):707–11. doi: 10.1016/j.eururo.2019.01.009 30665814

[49] Hofman P Ilie M Lassalle S Long E Bence C Butori C. PD1/PD-L1 Immunohistochemistry in Thoracic Oncology: Where Are We? Ann Pathol (2017) 37(1):39–45. doi: 10.1016/j.annpat.2016.12.006 28159404

[50] Herter-Sprie GS Koyama S Korideck H Hai J Deng J Li YY. Synergy of Radiotherapy and PD-1 Blockade in Kras-Mutant Lung Cancer. JCI Insight (2016) 1(9):e87415. doi: 10.1172/jci.insight.87415 27699275 PMC5033933

[51] Antonia SJ Villegas A Daniel D Vicente D Murakami S Hui R. Overall Survival With Durvalumab After Chemoradiotherapy in Stage III NSCLC. N Engl J Med (2018) 379(24):2342–50. doi: 10.1056/NEJMoa1809697 30280658

[52] Gourd E. Durvalumab Boosts Progression-Free Survival in NSCLC. Lancet Oncol (2018) 19(1):e11. doi: 10.1016/S1470-2045(17)30895-1 29175147

[53] Gray JE Villegas A Daniel D Vicente D Murakami S Hui R. Three-Year Overall Survival With Durvalumab After Chemoradiotherapy in Stage III NSCLC-Update From PACIFIC. J Thorac Oncol (2020) 15(2):288–93. doi: 10.1016/j.jtho.2019.10.002 PMC7244187 31622733

[54] Goto T. Radiation as an In Situ Auto-Vaccination: Current Perspectives and Challenges. Vaccines (Basel) (2019) 7(3):100. doi: 10.3390/vaccines7030100 PMC6789649 31455032

[55] Itamura H Ohguri T Yahara K Nakahara S Kakinouchi S Morisaki T. Pembrolizumab-Induced Radiation Recall Pneumonitis After the Resolution of Typical Asymptomatic Radiation Pneumonitis. J UOEH (2020) 42(3):261–6. doi: 10.7888/juoeh.42.261 32879190

[56] Teng F Li M Yu J. Radiation Recall Pneumonitis Induced by PD-1/PD-L1 Blockades: Mechanisms and Therapeutic Implications. BMC Med (2020) 18(1):275. doi: 10.1186/s12916-020-01718-3 32943072 PMC7499987

[57] Tubin S Popper HH Brcic L. Novel Stereotactic Body Radiation Therapy (SBRT)-Based Partial Tumor Irradiation Targeting Hypoxic Segment of Bulky Tumors (SBRT-PATHY): Improvement of the Radiotherapy Outcome by Exploiting the Bystander and Abscopal Effects. Radiat Oncol (2019) 14(1):21. doi: 10.1186/s13014-019-1227-y 30696472 PMC6352381

[58] Yin L Xue J Li R Zhou L Deng L Chen L. Effect of Low-Dose Radiation Therapy on Abscopal Responses to Hypofractionated Radiation Therapy and Anti-PD1 in Mice and Patients With Non-Small Cell Lung Cancer. Int J Radiat Oncol Biol Phys (2020) 108(1):212–24. doi: 10.1016/j.ijrobp.2020.05.002 32417411

[59] Barsoumian HB Ramapriyan R Younes AI Caetano MS Menon H Comeaux NI. Low-Dose Radiation Treatment Enhances Systemic Antitumor Immune Responses by Overcoming the Inhibitory Stroma. J Immunother Cancer (2020) 8(2):e000537. doi: 10.1136/jitc-2020-000537 33106386 PMC7592253

[60] Golden EB Chhabra A Chachoua A Adams S Donach M Fenton-Kerimian M. Local Radiotherapy and Granulocyte-Macrophage Colony-Stimulating Factor to Generate Abscopal Responses in Patients With Metastatic Solid Tumours: A Proof-of-Principle Trial. Lancet Oncol (2015) 16(7):795–803. doi: 10.1016/S1470-2045(15)00054-6 26095785

[61] Ball D Mai GT Vinod S Babington S Ruben J Kron T. Stereotactic Ablative Radiotherapy Versus Standard Radiotherapy in Stage 1 Non-Small-Cell Lung Cancer (TROG 09.02 CHISEL): A Phase 3, Open-Label, Randomised Controlled Trial. Lancet Oncol (2019) 20(4):494–503. 30770291 10.1016/S1470-2045(18)30896-9

[62] Lieverse RIY Van Limbergen EJ Oberije CJG Troost EGC Hadrup SR Dingemans AC. Stereotactic Ablative Body Radiotherapy (SABR) Combined With Immunotherapy (L19-IL2) Versus Standard of Care in Stage IV NSCLC Patients, ImmunoSABR: A Multicentre, Randomised Controlled Open-Label Phase II Trial. BMC Cancer (2020) 20(1):557. doi: 10.1186/s12885-020-07055-1 32539805 PMC7296663

[63] Palma DA Olson R Harrow S Gaede S Louie AV Haasbeek C. Stereotactic Ablative Radiotherapy Versus Standard of Care Palliative Treatment in Patients With Oligometastatic Cancers (SABR-COMET): A Randomised, Phase 2, Open-Label Trial. Lancet (2019) 393(10185):2051–8. doi: 10.1016/S0140-6736(18)32487-5 30982687

[64] Kulzer L Rubner Y Deloch L Allgauer A Frey B Fietkau R. Norm- and Hypo-Fractionated Radiotherapy Is Capable of Activating Human Dendritic Cells. J Immunotoxicol (2014) 11(4):328–36. doi: 10.3109/1547691X.2014.880533 24512329

[65] Deng L Liang H Burnette B Beckett M Darga T Weichselbaum RR. Irradiation and Anti-PD-L1 Treatment Synergistically Promote Antitumor Immunity in Mice. J Clin Invest (2014) 124(2):687–95. doi: 10.1172/JCI67313 PMC3904601 24382348

[66] Frey B Rubner Y Wunderlich R Weiss EM Pockley AG Fietkau R. Induction of Abscopal Anti-Tumor Immunity and Immunogenic Tumor Cell Death by Ionizing Irradiation - Implications for Cancer Therapies. Curr Med Chem (2012) 19(12):1751–64. doi: 10.2174/092986712800099811 22414083

[67] Twyman-Saint Victor C Rech AJ Maity A Rengan R Pauken KE Stelekati E. Radiation and Dual Checkpoint Blockade Activate Non-Redundant Immune Mechanisms in Cancer. Nature (2015) 520(7547):373–7. doi: 10.1038/nature14292 PMC4401634 25754329

[68] Jing H Hettich M Gaedicke S Firat E Bartholoma M Niedermann G. Combination Treatment With Hypofractionated Radiotherapy Plus IL-2/Anti-IL-2 Complexes and Its Theranostic Evaluation. J Immunother Cancer (2019) 7(1):55. doi: 10.1186/s40425-019-0537-9 30808414 PMC6390578

[69] Curti B Crittenden M Seung SK Fountain CB Payne R Chang S. Randomized Phase II Study of Stereotactic Body Radiotherapy and Interleukin-2 Versus Interleukin-2 in Patients With Metastatic Melanoma. J Immunother Cancer (2020) 8(1):e000773. doi: 10.1136/jitc-2020-000773 32467299 PMC7259841

[70] Seung SK Curti BD Crittenden M Walker E Coffey T Siebert JC. Phase 1 Study of Stereotactic Body Radiotherapy and Interleukin-2–Tumor and Immunological Responses. Sci Transl Med (2012) 4(137):137ra74. doi: 10.1126/scitranslmed.3003649 22674552

[71] van den Heuvel MM Verheij M Boshuizen R Belderbos J Dingemans AM De Ruysscher D. NHS-IL2 Combined With Radiotherapy: Preclinical Rationale and Phase Ib Trial Results in Metastatic Non-Small Cell Lung Cancer Following First-Line Chemotherapy. J Transl Med (2015) 13:32. doi: 10.1186/s12967-015-0397-0 25622640 PMC4320467

[72] Rekers NH Zegers CM Germeraad WT Dubois L Lambin P. Long-Lasting Antitumor Effects Provided by Radiotherapy Combined With the Immunocytokine L19-Il2. Oncoimmunology (2015) 4(8):e1021541. doi: 10.1080/2162402X.2015.1021541 26405576 PMC4570128

[73] Zegers CM Rekers NH Quaden DH Lieuwes NG Yaromina A Germeraad WT. Radiotherapy Combined With the Immunocytokine L19-IL2 Provides Long-Lasting Antitumor Effects. Clin Cancer Res (2015) 21(5):1151–60. doi: 10.1158/1078-0432.CCR-14-2676 25552483

[74] Gurbatri CR Lia I Vincent R Coker C Castro S Treuting PM. Engineered Probiotics for Local Tumor Delivery of Checkpoint Blockade Nanobodies. Sci Transl Med (2020) 12(530):eaax0876. doi: 10.1126/scitranslmed.aax0876 32051224 PMC7685004

[75] Doi T Piha-Paul SA Jalal SI Saraf S Lunceford J Koshiji M. Safety and Antitumor Activity of the Anti-Programmed Death-1 Antibody Pembrolizumab in Patients With Advanced Esophageal Carcinoma. J Clin Oncol (2018) 36(1):61–7. doi: 10.1200/JCO.2017.74.9846 29116900

[76] Zhao X Kong Y Zhang L. Anti-PD-1 Immunotherapy Combined With Stereotactic Body Radiation Therapy and GM-CSF as Salvage Therapy in a PD-L1-Negative Patient With Refractory Metastatic Esophageal Squamous Cell Carcinoma: A Case Report and Literature Review. Front Oncol (2020) 10:1625. doi: 10.3389/fonc.2020.01625 33014817 PMC7493754

[77] Kang J Demaria S Formenti S. Current Clinical Trials Testing the Combination of Immunotherapy With Radiotherapy. J Immunother Cancer (2016) 4:51. doi: 10.1186/s40425-016-0156-7 27660705 PMC5028964

[78] Crittenden M Kohrt H Levy R Jones J Camphausen K Dicker A. Current Clinical Trials Testing Combinations of Immunotherapy and Radiation. Semin Radiat Oncol (2015) 25(1):54–64. doi: 10.1016/j.semradonc.2014.07.003 25481267 PMC4640687

